# The Cytoskeleton—A Complex Interacting Meshwork

**DOI:** 10.3390/cells8040362

**Published:** 2019-04-18

**Authors:** Tim Hohmann, Faramarz Dehghani

**Affiliations:** Institute of Anatomy and Cell Biology, Martin Luther University Halle-Wittenberg, Grosse Steinstrasse 52, 06108 Halle (Saale), Germany; tim.hohmann@medizin.uni-halle.de

**Keywords:** actin, microtubules, intermediate filaments, motility, migration, glioma, signaling

## Abstract

The cytoskeleton of animal cells is one of the most complicated and functionally versatile structures, involved in processes such as endocytosis, cell division, intra-cellular transport, motility, force transmission, reaction to external forces, adhesion and preservation, and adaptation of cell shape. These functions are mediated by three classical cytoskeletal filament types, as follows: Actin, microtubules, and intermediate filaments. The named filaments form a network that is highly structured and dynamic, responding to external and internal cues with a quick reorganization that is orchestrated on the time scale of minutes and has to be tightly regulated. Especially in brain tumors, the cytoskeleton plays an important role in spreading and migration of tumor cells. As the cytoskeletal organization and regulation is complex and many-faceted, this review aims to summarize the findings about cytoskeletal filament types, including substructures formed by them, such as lamellipodia, stress fibers, and interactions between intermediate filaments, microtubules and actin. Additionally, crucial regulatory aspects of the cytoskeletal filaments and the formed substructures are discussed and integrated into the concepts of cell motility. Even though little is known about the impact of cytoskeletal alterations on the progress of glioma, a final point discussed will be the impact of established cytoskeletal alterations in the cellular behavior and invasion of glioma.

## 1. Introduction

A single animal cell has the ability to adapt its shape in response to environmental confinements or chemical cues, to move through tissues (artificial and in vivo, including narrow spaces), and to divide. These processes are all, at least in part, orchestrated by the (dis-)assembly of cytoskeletal proteins. The cytoskeleton is made up of three major types of proteins, as follows: Tubulin, actin, and proteins forming intermediate filaments. These cytoskeletal proteins differ not only in their chemical structure, but also in the type of filaments and structures they form, ranging from fast assembling dendritic actin networks of the lamellipodium, capable of generating forces necessary for cell movement over single microtubule filaments as transport structures, to intermediate filaments capable of promoting or inhibiting cell movement and stabilizing the cell against large stress.

To form a myriad of different cytoskeletal structures, as it is observed in animal cells, the cytoskeletal meshwork does not only need different components with different properties and functions, but also a tight and precise regulation of (dis-)assembly of its components, the respective local regulation of (dis-)assembly-factors, and interactions between the actin, microtubule, and intermediate filament networks.

The ability of cells to migrate is of special interest in glioma spreading, as the success of glioma treatment is crucially coupled to the question of whether a recurrent tumor will arise or not, as resection is successful only if the tumor is completely removed. Consequently, recurrent tumor formation is considered to be the main reason of tumor morbidity [1]. Hence, targeting the migratory machinery in gliomas can be a promising approach for the containment of metastasis. For successful targeting of glioma migration, a broad and detailed knowledge of the cytoskeletal architecture and its alterations is necessary.

Here we provide an overview of different cytoskeletal filaments, including actin, microtubules, and intermediate filaments, their (dis-)assembly, interactions, and function in motility and shape changes of healthy cells. Afterwards, cytoskeletal alterations in glioma and their impact on their migratory behavior are discussed.

## 2. Actin Regulation and Structure

In cells, actin occurs in two distinct states, as follows: The monomeric G-actin and filamentous F-actin. The modulation of the actin cytoskeleton is regulated by the balance of globular G- and polymeric F-actin and by actin associated proteins [2]. The actin cytoskeleton forms a network consisting of polarized filaments that are mostly associated with force generation necessary for movement, focal adhesion, and shape changes. In the following we describe the building blocks of actin filaments, the assembly and disassembly of filaments, their kinetics, regulation, as well as filament bundles, network structures, and their mechanical properties. A summary of all mentioned actin binding or associated proteins and their function can be found in Table 1.

### 2.1. Actin Filaments

**Figure 1:**
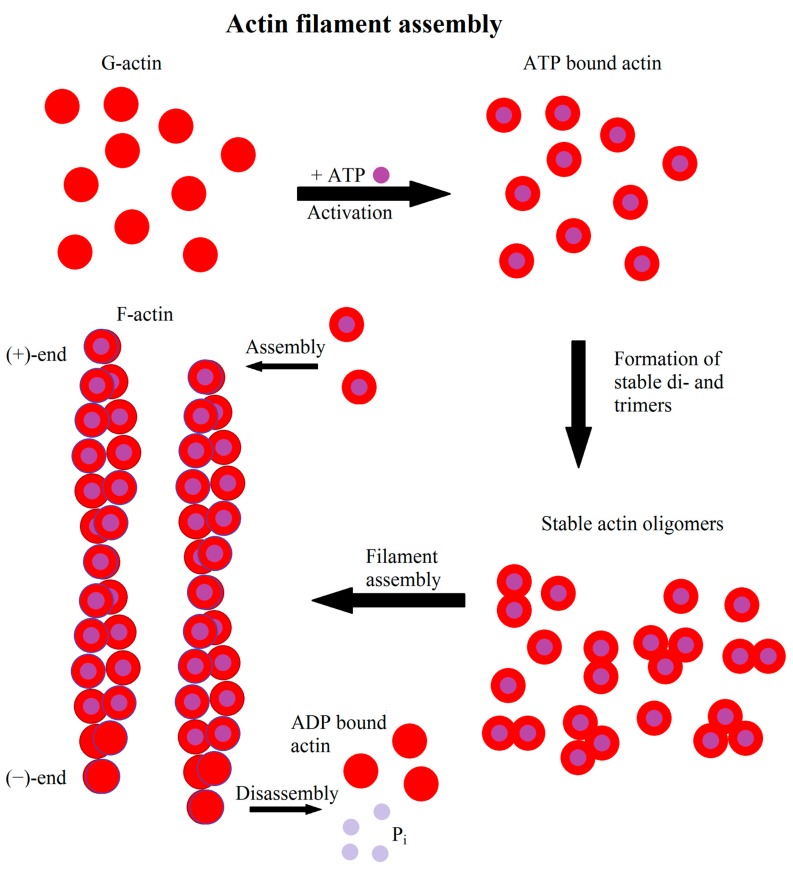
Scheme of actin filament formation. First G-actin binds to ATP. Afterwards, it forms stable di- or trimers and, finally, filaments elongate by addition of monomers. Hydrolysis of ATP to ADP leads to a distinction between the fast growing (+)-end and the slower growing or dissociating (−)-end.

Actin filaments are, in contrast to intermediate filaments and microtubules, semi-flexible filaments, forming dendritic or cross-linked structures. Semi-flexible means that the persistence length of a single filament is in the order of the filament length, where the persistence length is the length scale on which the correlation between two tangents along the filament drops to 1/e [3]. As a semi-flexible polymer, actin filaments are actively bent by thermal fluctuations, thus generating additional resistance to forces stretching the filament. Actin itself is considered the most dynamic of the three cytoskeletal proteins capable of strong structural changes in the time scale of minutes, thus determining the shape of a cell. A single actin filament consists of actin monomers, called globular actin (G-actin). Under nearly physiological conditions G-actin polymerizes to asymmetric helical structures, filamentous actin (F-actin), with a typical length of 6–7 µm in in vitro studies [4]. The nucleation kinetics is mostly limited by the generation of dimers and trimers [5]. Having reached the trimer state, filament nucleation increases rapidly, but in dependence of the available G-actin pool [6] (Figure 1). The resulting actin filaments have a right handed helical structure. G-actin is polarized, therefore F-actin is polarized as well, with the less dynamic side termed as the (−)-end and the more dynamic (+)-end having a ten times higher polymerization rate than the (−)-end [6]. As actin is an ATPase the (+)- and (−)-ends can also be distinguished by their ATP/ADP status, especially if the growth at the (−)-end is inhibited further. Thus the (+)-end contains higher amounts of ATP bound actin while the (−)-end contains more ADP bound actin.

### 2.2. Profilin

As F-actin is capable of forming spontaneously above a certain critical concentration of G-actin (≈0.1 µM) a precise cofactor-driven control of actin polymerization is necessary. One such control element is profilin. Profilin is an actin binding protein that regulates actin homeostasis [7,8] by inhibiting the spontaneous formation of actin di- and trimers, but it also catalyzes the transition from ADP- to ATP-actin [7]. Profilin bound G-actin can be used for the construction of actin filaments if nucleation factors like the Arp2/3 (actin-related-proteins 2/3) complex or formins are present [9]. Interestingly, if formins and profilin are present, free actin elongation can be increased by a factor of up to 9 [10,11]. Additionally, profilin was shown to inhibit polymerization at the (−)-end of actin [6].

### 2.3. Dendritic Actin Networks

Besides the quasi-linear actin filaments there are dendritic actin networks, formed by the Arp2/3 complex [12]. These networks are usually formed at the cell front on a short time scale [13] and, thus, its regulation is of high importance. The generation of a dendritic actin network starts from a so called primer, an existing actin filament at which Arp2/3 binds to its side [9,14]. Arp2/3 is, amongst others, activated by members of the WASP family [6,15]. For the generation of a dense dendritic network not only nucleation factors, but also capping proteins are needed to restrict the elongation of the actin (+)-ends [14,16,17,18]. If capping proteins are present the Arp2/3 complex can generate multiple networks originating from different actin filaments that are able to merge and generate forces near the cell membrane [14,17]. The number of nodes is important for the mechanical properties of the generated network and consequently the elastic modulus scales with the mesh size M by 1/M^4^. In general the dendritic network behaves visco-elastic, meaning it is mainly elastic on small time scales (<1 min) and viscous on longer time scales (>10 min) [14,17].

### 2.4. Non-Muscle Myosin

Another important molecule class is the myosin family. Here we will focus exclusively on the non-muscle myosin. Myosin is responsible for the contractility of anti-parallel actin structures using ATP hydrolysis as the energy supply [19,20]. These contractile structures are mainly responsible for the retraction of the cell rear for productive movement, but also for transmitting forces to the surrounding extra-cellular matrix.

Interestingly, myosin II motor activity alone is insufficient to produce contractility. Single myosin II hexamers are unipolar and thus ineffective in generating contractile forces [21], but when assembled into bipolar mini-filaments they are highly processive and capable of generating forces by pulling on anti-parallel actin filaments [22]. Myosin II can be activated via phosphorylation of the regulatory light chain (RLC) or activation of myosin light chain kinases (MLCK). RLC is activated via Rho-associated protein kinase (ROCK) or citron kinases (both activated by RhoA) and MLCK by Ca^2+^ [23]. After RLC phosphorylation myosin is capable of generating contractile forces [24,25]. Another type of regulation works via the phosphorylation of the myosin heavy chain, utilizing myosin heavy chain kinases (MHCK), casein kinase II (CKII), or protein kinase C (PKC), inhibiting mini-filament assembly or dissociating existing mini-filaments [26,27,28,29]. The switch between those two activation states influences the contractility of the respective acto-myosin network. Consequently, regulation of myosin strongly impacts organization and properties of contractile actin structures, as discussed below.

### 2.5. Cross-Linked Actin Networks and Actin Bundles

Despite the already mentioned dendritic actin structures, there are actin bundles and networks linked together by cross-linkers. Cross-linkers are molecules that connect single actin filaments either transiently or non-transiently and are either passive (e.g., scruin, fascin, α-actinin, filamin, or fimbrin) or active (myosin). Cross-linked actin bundles and networks largely control shape, mechanical integrity, and contractility of a cell [30,31,32]. Generally, cross-linkers do not influence actin assembly (except for Arp2/3) [32,33,34,35]. Cross-linkers bind actin filaments based on their own size and the position of their binding-sites in different distances, ranging from 10 nm for fimbrin to 160 nm for filamin, and thus determine the density of the resulting actin structure [36,37]. Additionally, the speed of actin polymerization influences the presence of cross-linkers in the resulting network, supposedly by crowding effects, thus excluding larger cross-linkers like α-actinin in quickly polymerizing filaments [33]. If the formed actin structures are subject to a force that acts on a longer time scale than the binding time of the cross-linkers itself, a reorganization of cross-linkers and a subsequent shape change of the bundle occurs [38]. This time scale depends on the type of cross-linker and its binding and unbinding time, which can be in the order of seconds [39,40]. Consequently, cross-linked actin is elastic on short and viscous on long time scales [38] and the presence of cross-linkers generally increases the elastic part of the visco-elastic answer to external stress.

If actin filaments are bundled by cross-linkers the filaments inside the bundle can either be oriented in parallel or anti-parallel, meaning that (+)-ends of neighboring filaments are pointing in the same or the opposite direction. Parallel actin bundles are found amongst others in filopodia [18,41], while anti-parallel bundles are mostly found in stress fibers.

Two mechanisms were proposed to explain the generation of parallel actin bundles. The first mechanism involves the Arp2/3 complex-dependent elongation of filaments in the absence of capping proteins, so that free growing (+)-ends transition into bundles via electro-static interactions between filaments [42]. Here, the geometric constraints and the angle at which filaments make contact to each other determine whether parallel or anti-parallel bundles are formed [42]. Notably, geometric constraints refer to all spatial limitations, such as the available free space, steric effects, etc., that potentially affect the final organization. Parallel bundles are then stabilized via cross-linkers, such as fascin. The second mechanism is formin dependent [43]. Thereby the FH1 (formin homology) domain of formin functions as a ring structure capturing profilin bound actin molecules, while the FH2 domain interacts with the (+)-end of the filament [44,45]. Some members of the formin family also move from the end of the filament into the middle, additionally functioning as cross-linkers to stabilize the generated structure [10,46,47]. As formins do not necessarily bundle filaments, further proteins, such as cross-linkers or Ena/VASP, are also involved in formin dependent bundle formation. Ena/VASP is a protein family associated with anti-capping function and elongation, but has no nucleation activity on its own [48].

In contrast to parallel bundles, anti-parallel bundles are mostly connected with classical cross-linkers and the motor-protein myosin, which has the ability to actively move antiparallel fibers relative to each other. As with parallel bundles, anti-parallel bundles are stabilized by cross-linkers favoring this configuration, like α-actinin or fimbrin [49,50,51]. Through the activation of myosin anti-parallel bundles are pre-stressed, leading to either a contraction or dissociation of the bundle [52,53]. Without further cross-linkers, anti-parallel bundles containing myosin first contract strongly and later disassemble [54].

The mechanical properties of (anti-)parallel bundles depends on the type and density of cross-linkers and, thus, on the compactness of the bundle and whether the bundle allows the sliding of single filaments. For non-cross-linked bundles the persistence length scales with the number of filaments while for cross-linked bundles that allow no filament sliding it scales with the number of filaments squared.

### 2.6. ADF/Cofilin Induced Actin Disassembly

As most of the actin structures are stable in time, cells need a mechanism to induce actin disassembly to adapt to environmental cues. One such mechanism is governed by the actin binding ADF/cofilin protein family, capable of disassembling and fragmenting actin filaments, but incapable of altering the polymerization rate [55,56]. The efficiency of ADF/cofilin depends on its binding state to actin filaments. Filaments that are fully decorated with ADF/cofilin are stabilized while partially decorated filaments fragment faster [57,58,59]. The induction of fragmentation is likely caused by a reduced persistence length of the filament, that can drop to ≈20% of its initial value through ADF/cofilin binding, locally generating increased mechanical stress [58,59,60]. ADF/cofilin preferentially binds to the (−)-end of actin filaments (ADP bound actin) [61], but binding to ATP bound actin favors its transition from ATP to ADP bound actin and, thus, accelerates filament dissociation [62]. The preference for older ADP bound actin implies that ADF/cofilin disassembly mostly affects inactive compartments of the actin network [55,56,63,64]. As ADF/cofilin cannot bind to the free (+)-end it can only fully saturate actin filaments if the (+)-end is bound by capping proteins and is not capable of further elongation [65]. With the exception of fascin, cross-linkers and tension reduce the efficiency of ADF/cofilin fragmentation [34,66,67,68]. Furthermore, ADF/cofilin is present in high concentrations at the leading edge in dendritic networks, fragmenting links generated by Arp2/3 and the actin filaments itself, generating more free (+)-ends [69].

### 2.7. Actin Structures Inside the Cell

**Figure 2:**
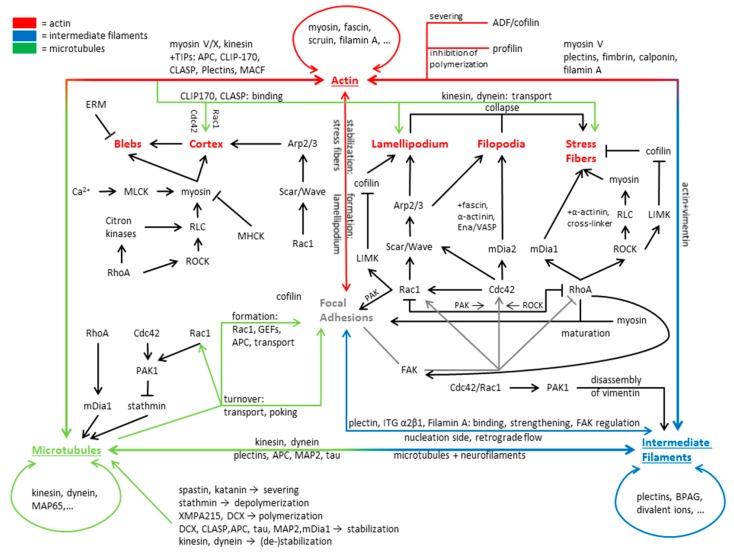
Illustration of actin, microtubule, and intermediate filament signaling, with focus on migration associated structures and signaling cascades.

Looking at a motile cell the net movement is the result of multiple, mostly actin-dependent, processes, as follows: Formation of protrusions in direction of motion, subsequent adhesion to the substrate and loss of adhesion on the rear of the cell, followed by rear-contraction (reviewed in [70,71,72,73]). These processes are governed by sub-cellular structures, like filopodia and the lamellipodium governing cell motion, while stress fibers and the cortex secure mechanical stability and contractility. Further types of protrusions are so called blebs, which are capable of regulating cell movement independently of filopodia and the lamellipodium. The interactions of actin discussed here and its structural and functional integration with microtubules and intermediate filaments are summarized in Figure 2.

#### 2.7.1. The Lamellipodium

**Figure 3:**
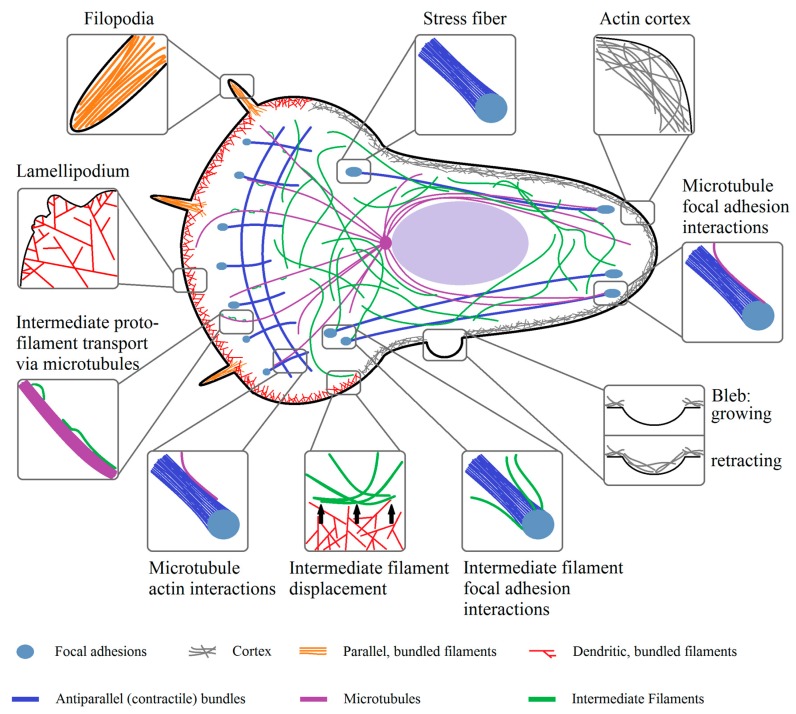
Organizational structures of actin, microtubules, and intermediate filaments inside of a cell and their physical interactions. Notably, all three cytoskeletal proteins interact directly with each other.

The lamellipodium (Figure 3) is a flat structure mainly associated with cell movement, formed by the polymerization of actin at the cell front [13,74], while it is depolymerized at the back of the lamellipodium by ADF/cofilin refilling the G-actin pool [75]. The continuing (de-)polymerization of the whole network creates a treadmilling effect and a retrograde actin flow in the cell [76,77], which is enhanced in some cell types by myosin induced depolymerization at the back of the lamellipodium [78]. Any flow originating from the contraction of the rear via stress fibers generates a flow of opposite direction [77]. The forces generated by actin polymerization in the lamellipodium are up to a few hundred pN/µm [79]. The most important factor for the generation of the lamellipodium is the intrinsically inactive Arp2/3 complex that becomes activated by the Scar/WAVE complex in an activation process by the small Rho GTPase Rac1 [80]. Arp2/3 nucleates a new actin filament at the site of existing filaments [80]. For a three dimensional environment, N-WASP (and not WAVE) was shown to activate Arp2/3 and Rac1 was not found to be strongly localized at the cell front [81,82]. Actin growth is further promoted by the presence of members of the Ena/VASP family accumulating at the lamellipodial tip, promoting further actin elongation and preventing capping [83,84]. Despite the active Arp2/3 complex, a capping protein is needed as well to limit the elongation of single filaments [16,85] so they remain productive and do not form bundles with other uncapped filaments or buckle under the load [86]. For the generation of a stable dendritic network, it is cross-linked by proteins such as cortactin [87]. As the described regulation by Rac1 would result in a constant growth of the lamellipodium, it has to be restricted by a negative feedback loop. One possible mechanism is via the protein arpin, which inhibits Arp2/3 activity in the lamellipodium [88]. It has been postulated that arpin is recruited by Rac1 [88]. Thus, it seems possible that Rac1 activation initiates lamellipodium growth via quick Arp2/3 recruitment and successive actin polymerization and later inhibits its growth via recruitment of arpin. A high turnover rate of arpin or significantly higher concentration might be necessary to inactivate Arp2/3 [89]. A proof for this kind of hypothesis is yet lacking. Despite the actin dynamics, the lamellipodium is also influenced by the cell membrane and its surface tension [90]. A higher membrane surface tension led to a more oriented actin filament polymerization while a lower tension resulted in more protrusions [90], probably related to the finite forces generated by the lamellipodium. Regarding the mechanical properties of the lamellipodium, it has to be noted that myosin was observed to be present at the rear of the lamellipodium, explaining why the lamellipodium is elastic on short and viscous on long time scales [78,91].

Due to Arp2/3 the actin in the lamellipodium is connected to a dendritic structure [92]. Interestingly, an analysis of cell speed relative to the actin orientation in the lamellipodium could demonstrate that faster cells have filaments that are not exactly oriented in the direction of movement and the parallel orientation of filaments is associated with slower movement [93].

#### 2.7.2. Filopodia

Further structures associated with cell motility are filopodia (Figure 3). Filopodia are associated with a sensory function in neurons [94], but do not seem necessary for migration, as the fast moving corneal keratocytes do not possess filopodia in two dimensions and forces generated by filopodia are significantly smaller than those generated by the lamellipodium [95]. In other systems there might be a role for filopodia in migration, e.g., in three dimensional systems [96]. Filopodia form a structure consisting of parallel actin bundles, with their (+)-ends pointing in direction of the cell membrane [97]. This orientation is established via formins (e.g., FMNL2) and Ena/VASP, both being capable of maintaining a prolonged actin polymerization [98]. Some of these formins, like mDia2, can be activated by the small GTPase Cdc42 [99]. Cdc42 is also capable of activating N-WASP and thus Arp2/3, leading to filopodia formation [100]. A common model for filopodia initiation suggests that actin polymerization occurs in the presence of activated Arp2/3 and without capping proteins forming actin bundles [98]. Nevertheless, Arp2/3 does not seem to be necessary for filopodia initiation in adherent cells [101]. A further model of filopodia initiation states that filopodia are initiated by clusters of activated formins near the plasma membrane, nucleating and elongating actin filaments [98]. For both models, subsequent further elongation via formins (e.g., mDia2) and Ena/VASP and stabilization and bundling with cross-linkers, like fascin, generates “mature” filopodia [102]. Besides their role in cell movement, filopodia initiate cell-cell contacts, transmit cell-cell-signals, and respond to the mechanical properties of their surroundings [103]. Interestingly, when filopodia are retracted to the cell the myosins II,V, and VI are not involved in this process [104]. This leads to the idea that only actin (de-)polymerization and changes in the cortex are responsible for filopodia dynamics.

#### 2.7.3. Stress Fibers

Another type of actin related structures are stress fibers (Figure 3) that are neither present in filopodia nor in the lamellipodium. Stress fibers are formed from bundles of anti-parallel actin filaments containing myosin II or parallel filaments [105]. Stress fibers are assembled bundles of 10–30 actin filaments [106], cross-linked by α-actinin in a bipolar fashion, and linked to focal adhesions [105,107]. Focal adhesions are binding sites that connect the cell to the substrate. Contractile stress fibers are one of the main contributors to cell contractility in many animal cells. As the contractility of these stress fibers is regulated by myosin [108], regulation of stress fiber contractility is in many ways similar to the regulation of myosin activity discussed before. In non-motile cells, stress fibers are usually thick and comparably stable, while motile cells typically contain fewer less pronounced fibers with a higher dynamic [109]. Actin and myosin are the two principal constituents of contractile stress fibers, while non-contractile ones do not contain myosin [110]. Despite these components, stress fibers contain actin binding proteins and focal adhesion-associated proteins binding and unbinding in quick succession [110,111,112,113]. The molecules found in stress fibers include cross-linkers, such as α-actinin [114], which does not only function to stabilize the bundle but is also associated with kinases and signaling proteins and, thus, functioning as a signaling mediator [115,116]. Stress fibers can contain further cross-linkers, like fascin, filamin, and paladin, but their precise role despite bundling remains elusive [117,118,119]. One hypothesis states that these proteins function as basis for regulation of cytoskeletal dynamics as, for example, paladin interacts with profilin and VASP [120,121]. Further molecules of e.g., the calponin, tropomyosin, caldesmon family, and others, are found in stress fibers and are all suggested to be part of the cytoskeletal and/or stress fiber regulation [114,122,123,124,125]. Generally speaking, stress fiber formation has been directly associated with an activation of the formin mDia1 and the small Rho GTPase RhoA, activating ROCK [126,127]. The formin favors prolonged actin polymerization of parallel filaments important for dorsal stress fibers [110,128]. In contrast ROCK activates the LIM kinase (LIMK), which inhibits ADF/cofilin induced filament severing [129], and additionally, ROCK activates myosin, favoring stress fiber formation [23,105]. Nevertheless, both the ROCK and formin mechanisms are necessary for the formation of contractile stress fibers [109]. Two other Rho GTPases, Cdc42 and Rac1, act in more indirect ways via the induction of lamellipodial growth via Arp2/3 (Rac1) and filopodia formation via the formin mDia2 (Cdc42) [99,130,131,132]. Collapse of both filament types can function as seeds for either transversal or ventral stress fibers.

Since stress fibers vary in their morphology, molecular signature, and association with focal adhesions, the four following types of stress fibers can be distinguished: The perinuclear actin cap, transverse arcs/stress fibers, and dorsal and ventral stress fibers.

The three classes of contractile stress fibers are the ventral and transverse stress fibers and the perinuclear actin cap, all characterized by the presence of myosin II along the fibers. Even so, the myosin II spacing can change over time, indicating that contractile stress fibers are dynamic structures with non-uniform mechanical properties [133]. Measurements indicate that stress fibers have a stiffness of roughly 12 kPa, constant for strains up to 0.12 [134]. Perturbation of myosin in stress fibers reduces the elastic modulus to 8 kPa, indicating the importance of myosin II in contractile stress fibers [134]. Not surprisingly, the tensile elastic modulus increases from approximately 1.5 MPa to 104 MPa for strains approximating 2 [135]. Ventral stress fibers are oriented parallel to the direction of cell motion and connect adhesion sites of the cell, while transverse fibers are oriented perpendicular to the ventral fibers and are not directly connected to focal adhesions. Even so, transversal stress fibers can contribute to the overall contractility through their connection to dorsal stress fibers [110,136]. The formation of transversal stress fibers is dependent on Arp2/3 and myosin [110] and possess a periodic α-actinin-myosin pattern [137]. Transversal stress fibers form when the dendritic network collapses and is restructured by myosin [138,139]. Notably, simulations on the capability of myosin to generate contractile structures suggest that the presence of myosin and actin is sufficient to generate anti-parallel/contractile bundles, as these were found to be energetically favorable [140]. A further origin of both transversal and ventral fibers is the collapse of filopodia, which functions as a seed for stress fibers [141]. Additionally, ventral stress fibers can be formed from existing dorsal stress fibers and the attached transverse stress fibers, as well as by the fusion of two dorsal stress fibers [110,142]. Ventral stress fibers are also contractile actin-myosin bundles attached to focal adhesions at both ends, thus being directly part of the contractile machinery [143]. Due to their location at the rear of the cell and an orientation that is roughly in the direction of motion, they are part of the rear contraction, and thus associated with cell motility [73,144]. The third type of contractile stress fibers is the so-called perinuclear cap, consisting of stress fibers positioned above the nucleus, regulating the shape of the nucleus. Additionally, they are proposed to serve as a mechanical connection between the nucleus and the rest of the cell [145]. All of these contractile stress fiber types have in common that they are highly dependent on presence and activity of myosin and, thus, on tension. Consequently, myosin inhibition leads to the disassembly of these stress fibers [146].

In contrast to the other stress fiber types, dorsal stress fibers do not contain myosin II [110,111] and are anchored at focal adhesions at their distal ends [110,136]. The lack of myosin directly leads to the lack of contractility of dorsal stress fibers. It is proposed that these fibers consist of fast growing (+)-ends that face the cell periphery and more distant parts consisting of mixed polarity actin filaments [106,109]. Furthermore, paladin and Rac1 are seemingly essential for the formation of dorsal stress fibers. Paladin promotes fiber assembly via VASP recruitment [147,148]. Functionally, these stress fibers seem to be an anchor point for the assembly of the other stress fiber types and a link to focal adhesions [110,111]. It is supposed that dorsal stress fibers are generated via actin polymerization at emerging focal adhesions [110] and stabilized during retraction phases of the lamellipodium via coupling to emerging transverse stress fibers [111,138,149].

#### 2.7.4. Actin Cortex and Blebs

The last cytoplasmic structure described here is the actin cortex (Figure 3), which forms a contractile actin structure at the boarder to the plasma membrane. The cortex is a few hundred nanometer thick layer, consisting of a mixture of filament bundles and cross-linked filaments, with a mesh-size of approximately ≈50–150 nm [150], a thickness of 50–100 nm [151,152], and a distance to the cell membrane of less than 20 nm [151]. The cortex meshwork appears to be mainly isotropic, oriented in parallel to the plasma membrane, but some filaments are also oriented perpendicular to the membrane [153]. Despite actin filaments, the cortex contains a number of cross-linkers (e.g., fascin, actinin, filamin, etc.), myosin, proteins that control actin turnover (like profilin, cofilin), capping proteins, proteins of the ERM (ezrin, radixin, moesin) family, nucleating factors (like Arp2/3, the formin mDia1), and signaling molecules such as RhoGTPases, RhoGEFs (guanine exchange factors), and RhoGAPs (GTPase activating proteins) [154,155]. The two mentioned nucleating factors Arp2/3 and mDia1 were also found to be responsible for the majority of cortical F-actin generation [155,156], while the ERM proteins link the cortex to the membrane and can therewith transmit forces acting on the membrane and determine the cell shape [157,158,159]. Depletion of cofilin-1 or capping proteins in HeLa cells increased cortex thickness but reduced tension, implying a role for actin regulating proteins in cortical tension [160]. Mechanical properties of the cortex determine how the cell deforms in response to external forces. On time scales smaller than the remodulation time of the cortex it behaves elastic [161], with a cell type-dependent elastic modulus in the order of a few hundred to thousands of pascals [162,163]. On long time scales (>1min) the cortex behaves viscous because of the adaption to external forces via actin modulation, dissociation, and (un-)binding of cross-linkers [161]. If myosin is activated the cortex turnover times can be even lower [164,165], perhaps via direct disassembly or enhanced actin breakage [52,78]. Generally speaking, the behavior of the cortex is similar to that of glassy materials [166] and consistent with relaxations of three dimensional in vitro actin networks [167].

One of the main global and local properties of the cortex is its tension, which regulates the cell shape of single cells and tissues [168]. Several studies demonstrated that the cortex tension depends on the myosin activity and actin polymerization, with higher myosin activity and lower actin polymerization leading to an increased cortex tension [169]. A lower cortex tension is further associated with an increased protrusive activity of the cell, thus indirectly regulating cell motility [170]. Interestingly, local drops in cortex tension or cortex-membrane adhesion and local ruptures of the cortex can be the origin of so called blebs (Figure 3), a special, initially actin free, membrane protrusion [171]. Blebs can be initiated by any type of cortex weakening or loss of cortex-membrane adhesion if a given internal hydrostatic pressure threshold is reached [172,173]. Localized myosin contractions, promoting either cortex tearing or increasing local intracellular pressure, are some of the main sources of blebbing [174,175,176], but others are also discussed [177]. Thus, the activation of myosin via the already described activation by ROCK or MLCK are sufficient to induce bleb formation [178,179,180]. The progress of a bleb can be divided in three steps, as follows: Initiation, growth, and retraction. Initially, the growing bleb does not contain actin, but over time, when the bleb expands further, the actin cortex reassembles at the plasma membrane, stalling the bleb growth [157] up to the point of a full restoration where the generated contractile forces retract the bleb [181]. It has to be noted that bleb retraction does not always occur and in some motile cells blebs are stabilized and used as an alternative or additional mode of migration [178,179,180,182]. The expansion of blebs by actomyosin contraction induced pressures lasts 5–30 s, accompanied by a flow of cytosol into the bleb and a concomitant increase in surface area. The surface area is increased by a flow of lipids through the tearing of the membrane from the actin cortex [183]. The maximal bleb size is determined by the initial growth rate and the cortex re-polymerization time [184], both being dependent on cortex tension. The concept of tension inhibiting bleb expansion is further supported by the idea that the needed membrane unfolding is effectively resisting bleb expansion and, thus, slowing down the growth [182,185]. After full maturation the cortex is reconstructed and if the bleb is not stabilized via adhesions it is retracted by the re-established cortex via a myosin induced contraction [178,186].

#### 2.7.5. Nuclear Actin

For completeness it has to be mentioned that actin is not only present in the cytoplasm of eukaryotic cells, but also in the nucleus. As in the cytoplasm, nuclear actin exists in a monomeric and polymeric form [187]. Nuclear G-actin was shown to associate with all three RNA polymerases, participating in transcription initiation and elongation [188]. The exact function of G-actin in the transcription complex remains unclear, but G-actin levels need to be precisely tuned for normal translation [189] and cofilin is required for its elongation [190]. Contrary, stable actin filaments inhibit transcription [191]. Furthermore, actin is implied to affect the nuclear structure. During nuclear expansion at the mitotic exit, chromatin reorganizes depending on the transient formation of polymeric actin, in a seemingly cofilin dependent manner [192]. It is probable that nuclear actin regulates the structure of the nuclear envelope and the nucleus via interactions with the nuclear intermediate filaments lamin [193].

As mentioned, cell nuclei contain polymeric actin that can be generated inside the nucleus via actin nucleation factors [194]. These actin assembly factors include, amongst others, mDia1, Spire1/2, Fmn2, and Arp2/3 [195]. The presence of assembly factors underlines a functional role of polymeric actin and actin binding proteins. Initiation of DNA replication was demonstrated to require formin dependent nuclear actin polymerization [196]. Additionally, the loss of Spire1/2 and Fmn2 resulted in a less efficient clearance of DNA double strand breaks [197]. This is in agreement with two studies demonstrating an association of actin and Arp2/3 with sites of DNA damage and decreased damage repair after reduced Arp2/3 dependent actin nucleation [198,199]. For more information on the role of actin in the nucleus the interested reader is referred to other reviews [188,200].

## 3. Microtubules

**Figure 4:**
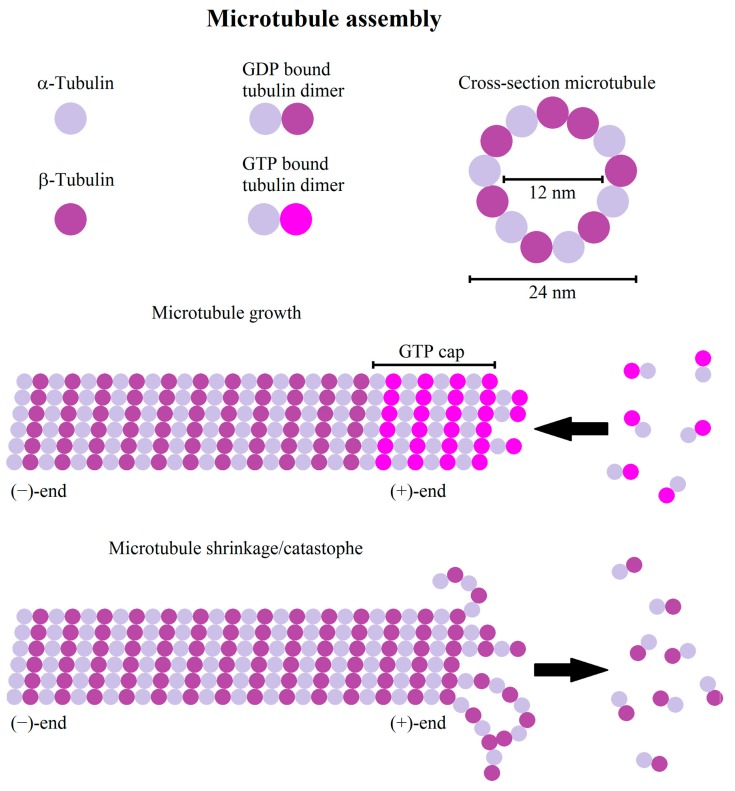
Scheme of microtubule formation and dynamic instability. Microtubules consist of α- and β-heterodimers, forming a hollow tube elongating by the addition of heterodimers, forming a GTP-cap at the (+)-end of the microtubule, protecting microtubules from shrinkage. If the (+)-end loses its GTP-cap it induces microtubule shrinkage.

Microtubules consist of α- and β-tubulin heterodimers forming hollow filaments, usually consisting of 13 protofilaments [201]. The microtubules are seeded by the microtubule organization center (MTOC), generally centrosomal, but in some types of differentiated cell non-radial microtubules are assembled by non-centrosomal MTOCs, for example at the Golgi apparatus [202,203,204]. MTOC, amongst others, contains γ-tubulin as a microtubule nucleator, as well as anchoring and adaptor proteins for attachment of microtubules [205]. Heterodimers interact at the MTOC with the γ-TURC, thus microtubules are nucleated and anchored with their (−)-ends to the MTOC [205]. Microtubules show a behavior called dynamic instability, characterized by a sudden switch from growing to growth arrest and/or quick depolymerization (termed catastrophe), followed by a new growth cycle [201] (Figure 4). A possible explanation for this behavior can be derived from the formation process of single protofilaments. During polymerization, GTP-bound tubulin heterodimers are bound to the (+)-end and normally hydrolyzed shortly after, but sometimes older parts of microtubules still contain GTP-bound tubulin heterodimers [206]. This leads to the following model: If, due to stochastic fluctuations or other perturbations, the (+)-end does not contain the more stable GTP-bound tubulin, it depolymerizes and is stabilized at older GTP-bound tubulin sites [206]. Stochastic fluctuations in the rate of microtubule growth and the stochastic nature of GTP hydrolysis lead to a dynamic GTP cap size [207], with the consequence that faster polymerizing microtubules have a larger GTP cap, resulting in less frequent catastrophe events [208]. The dynamic behavior of microtubules can be regulated by both intrinsic and extrinsic factors (see Table 2 for summary). Microtubule interacting proteins are either microtubule (+)-end-binding proteins (+TIP) or structural microtubule-associated proteins (MAP) interacting with microtubules along their length. These protein classes can have stabilizing or destabilizing effects, changing polymerization dynamics or severing microtubules. Important proteins belonging to the family of the +TIPs are CLASPs (cytoplasmic linker associated protein) and APC (adenomatous polyposis coli) [209,210] that suppress microtubule catastrophe events and promote rescue after catastrophe [211]. Part of the stabilizing effects of CLASPs arises from their capability to modulate interactions between microtubules and the cell cortex [212]. Further important families of +TIPs are the spectraplakins, binding both microtubules and actin [213] and EBs (end binding proteins). EBs are supposed to be a master regulator of +TIP recruitment (e.g., CLASP [214], APC, MACF1 (microtubule-actin crosslinking factor) [215]) and complex assembly [211], generally promoting persistent microtubule growth [216]. EBs are generally associated with an increased polymerization rate and reduced catastrophe number [217,218]. Despite these molecules that mainly (de-)stabilize microtubules, there is a bunch of proteins that sever microtubules, like spastin [219] or katanin [220], or influence depolymerization and polymerization, e.g., stathmin (favors depolymerization) [221] or XMPA215 (increases polymerization rate) [222]. Additionally, there are structural MAPs, like tau protein, MAP2, or DCX (doublecortin), that interact with the filament at its whole length and stabilize it [223,224] by reducing shrinkage speed, promoting filament growth, and reducing catastrophe frequency [223,224]. The effect of structural MAPs can also inhibit the effect of other microtubule associated proteins, as, for example, tau protein can inhibit the katanin induced severing [225]. A further important class of MAPs are the motor proteins kinesin and dynein, both serving as cargo transporters, exploiting the microtubule meshwork [226,227]. In general, kinesin motor proteins transport cargo to the (+)-end, while dynein moves to the (−)-end of microtubules [228,229], transporting diverse cargo types, including membrane components, signaling molecules, such as the small GTPases Rac and Cdc42 [230,231], but also intermediate filaments and their precursors [232,233,234], β-actin coding mRNA, and sub-units of the Arp2/3 complex [235,236]. Motor proteins possess not only a transport function but can stabilize or destabilize microtubules. For example, members of the kinesin-8, kinesin-13 family, or KinI kinesins can induce depolymerization, likely via the destabilization of the GTP cap [237] or the induction of kinks [238]. Despite their transport function and regulatory role in microtubule dynamics, some kinesins organize the microtubule network via the bridging of microtubules, thus favoring the generation of parallel arrays. For example, in neurons kinesin-5 and kinesin-12 are necessary for axonal outgrowth because of their cross-linking ability and the concomitant focus on the extension of microtubule arrays [239,240]. Additionally, kinesin-1 may be involved in this process by sliding filaments alongside each other [241]. A further important aspect regulating microtubule dynamics are the post-translational modifications modifying microtubule properties and affinities of MAPs [242]. Important modifications are, amongst others, tyrosination, glutamylation, and acetylation. Acetylation protects microtubules against repeated mechanical stress via an increased flexibility, but does not protect against depolymerization [243]. Additionally, some severing proteins, such as katanin, preferentially interact with acetylated tubulin [244]. Tyrosination affects the recruitment of microtubule interacting proteins, such as CLIP-170 or kinesin-1, that prefer detyrosinated microtubules, probably facilitating directional transport [245,246]. In contrast, spastin favors cleavage of detyrosinated microtubules [247]. Similar to tyrosination, glutamylation can also affect the interaction with microtubule associated proteins. Map2, tau, and kinesin-1 were reported to preferentially interact with those microtubules with up to three glutamates on their tail [245]. Similarly, both microtubule severing proteins, katanin and spastin, show an increased affinity for glutamylated microtubules [248,249]. Taken together, this data suggests that post translational modifications are important and finely tunable regulators of microtubule dynamics and, consequently, of cell behavior.

For regulation of MAPs, and microtubules in general, the family of Rho GTPases is of major significance. An important example is the stabilizing effect of RhoA, but not Rac1 and Cdc42, on microtubules via the RhoA effector mDia. mDia is capable of interacting with EB1 and APC, leading to a stabilization of microtubules via e.g., Kif4 [250,251]. Furthermore, active mDia induces the alignment of actin and microtubules [251,252]. Interestingly, stathmin action seems to negatively regulate the RhoA/ROCK activity [253], complementing the observation that Cdc42 and Rac1 phosphorylate stathmin via an activation of PAK (p21-activated kinases) [254,255]. In fact, Rac1 activation was demonstrated to decrease catastrophe events and increase microtubule growth time in cells via PAK [255,256]. Furthermore, both Rac1 and Cdc42 activate IQGAP1, interacting with Clip-170, likely providing a stabilization site for microtubule (+)-ends near the cortex [257]. The interactions of microtubules discussed here and their interactions with actin and intermediate filaments are summarized in Figure 2 and Figure 3.

Microtubules typically play a role as tracks for transport, as already mentioned, in spindle positioning during mitosis, migration (discussed below), and in cell shape control [258,259,260]. At the current point, the concept that microtubules control the balance between RhoA and Rac1 and thus influence cell shape and migration is favored over a direct mechanical participation for most cell types [261]. Even so, microtubules are relatively stiff polymers, when compared to actin [262], capable of generating forces of up to 3–4 pN during polymerization [263,264,265]. As a result, microtubules can deform membranes and resist compressional forces in such a way that they act as load bearing fibers in living cells via transversal re-enforcement by other cytoskeletal components [266,267]. Notably, if multiple microtubule filaments grow as a bundle the generated forces increase linearly with the number of microtubules per bundle [268]. Complementary to these observations, the load bearing capacities of microtubules is limited because compressional loads can induce catastrophe events [265,268], in line with the fact that most catastrophes are indeed induced at the cell edge [269,270] and the observed short-wavelength buckles near the boundary of adherent cells [170,271]. A further mechanism of force generation by microtubules is during the shrinkage phase. To actually transmit a force during shrinkage, the (+)-end needs to stay attached to its cargo. When the GTP cap is lost the microtubule protofilaments lose their lateral connection with neighboring protofilaments, bending backwards and forming ring-like shapes [272,273] (Figure 4). If cargo stays attached during this process, a single microtubule can exert forces of up to 30–65 pN [272,273], a magnitude larger than the pushing force [263].

Microtubules also play a key role during the separation of chromosomes during cell division [274]. Depolymerization of microtubules is believed to generate the needed forces to separate the sister chromatids [275]. This aspect will not be discussed in more detail here, but the interested reader may be referred to the following reviews: [274,275,276].

Furthermore, microtubules and actin are linked regarding their functional dynamics and structural organization. On the one hand there is an indirect co-regulation, as microtubules are able to locally regulate and are regulated by RhoGTPases and focal adhesions (see also chapter 5.2), but on the other hand, there are molecules interacting with microtubules and actin. One such molecule is APC, which stabilizes microtubules and nucleates actin filaments, with actin nucleation additionally favored by the formin mDia1 [209,277]. A further formin mDia2 that is capable of nucleating actin can also stabilize microtubules, independent of its nucleation function [278]. There are additional cross-linkers connecting actin and microtubule filaments, such as MACF1 and Arg [279,280]. Consequently, the actin and microtubule cytoskeleton cannot fully be regarded as decoupled systems, as they are not regulated independently and can even be connected physically.

## 4. Intermediate Filaments

**Figure 5:**
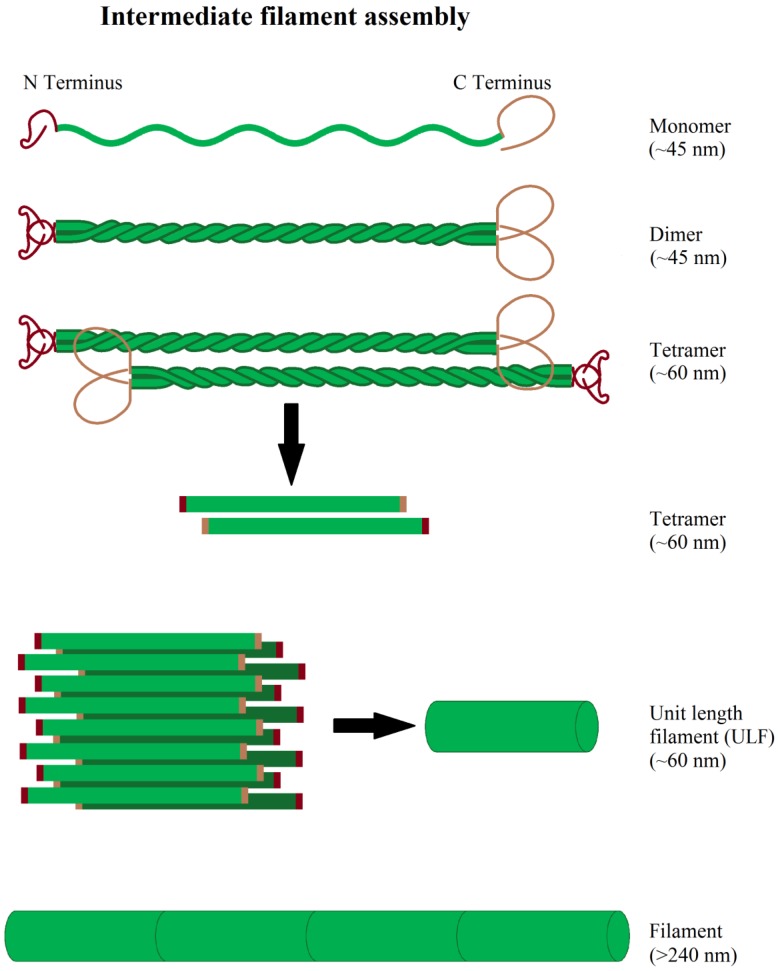
Illustration of intermediate filament assembly. Intermediate filaments arise from the monomers spiraling around each other to form dimers. Two dimers aggregate to a tetramer and eight tetramers to a unit length filament. Unit filaments form the final filament via end-to-end aggregation. Notably, this process is independent of ATP or GTP.

Intermediate filament-forming proteins are a large protein class, encoded by at least 70 genes, organizing filaments with a diameter of 10 nm. Intermediate filaments are grouped in 5 classes according to their structure and sequence homology. Thereby, the first four classes represent cytoplasmic intermediate filaments, while type V are nuclear filaments, so called lamins (lamin A/C, B1, B2). Type I and II are acidic and basic keratins, forming heteropolymers consisting of a mixture of the 54 different type I and II keratins, expressed in dependence of cell type and differentiation status [281]. In contrast, type III intermediate filaments are homopolymers of vimentin, desmin, peripherin, or glial fibrillary acidic protein (GFAP). Vimentin is mainly expressed in fibroblasts, endothelial cells, astrocytes; peripherin in neurons of the peripheral nervous system and desmin in muscle cells and GFAP mainly in astrocytes. Type IV intermediate filaments contain three neurofilament heteropolymers (NF-L/M/H), internexin, synemin, and nestin, mainly expressed in the cells of the nervous system. Nestin and synemin cannot form filaments on their own, but only in conjunction with other intermediate filament proteins [282,283]. Two further intermediate filaments, called filensin and phakinin, cannot be grouped into the mentioned five types. They are expressed in the lens epithelium, forming heteropolymers [284]. All cytoplasmic intermediate filaments have a similar monomer structure, consisting of a central α-helix with a non-helical structure at both of its ends [285]. Two monomers spiral around each other, forming a so-called “coiled-coil” dimer [286] and, subsequently, these dimers form unpolarized tetramers via antiparallel association and 8 tetramers form a cylindrical unit-filament [287]. The unit filaments aggregate further with other unit filaments at the time scale of minutes to form intermediate filaments [288] (Figure 5). After aggregation, the filaments undergo a compaction step during which the filament diameter shrinks to its final size of approximately 10 nm [289,290,291]. For nucleation and polymerization of intermediate filaments co-factors are not needed [292]. Intermediate filaments show a constant, but slow, subunit exchange along the whole filament, occurring at a rate of approximately 1 per 200 tetramers per hour in vitro for vimentin [293].

Inside the cell, cytoplasmic intermediate filaments form a dense meshwork that is mainly located in the perinuclear space, but also reaches the cortex [294]. The form and structure of the network depends on the type of intermediate filament. While keratin forms bundles and fibers that form only weakly connected networks [295], vimentin and desmin form highly connected networks with small mesh size and lamins generate filaments and fibers [296,297,298]. Near the cortex, intermediate filaments interact with focal adhesion sites, desmosomes, and hemidesmosomes, maintaining cell and tissue adhesion [299,300,301,302]. Conversely, desmosomes and focal adhesions function as centers for de novo intermediate filament formation [303]. Via their anchorage with the nuclear and plasma membrane, intermediate filaments form a scaffold for mitochondria, the Golgi apparatus, and further organelles and organize their location [304,305,306]. Due to its network structure and its ability to anchor organelles, intermediate filaments are often considered to be mechanical buffers [284,307,308]. This idea is supported by the single filament properties of intermediate filaments that can withstand deformations of up to 300% of their initial length without rupturing [309]. Looking at the elastic properties of intermediate filaments, they can be considered as flexible polymers with a persistence length of less than 1 µm [309]. Interestingly, intermediate filaments show a strongly increasing elastic modulus with increasing deformation (called strain hardening) [310]. Measurements of single cells and simulations could confirm an important role of intermediate filaments for the overall visco-elastic response of a cell [311,312,313].

Despite its function as a “mechanical buffer” and “organelle anchors” [284,307,308,314], intermediate filaments are highly dynamic components of the cytoskeleton, with multiple functions, including roles in apoptosis, migration, adhesion, and interactions with other cytoskeletal components. For fulfilling these functions, intermediate filaments need to form a defined network capable of (in-)direct interaction with its targets. Organizing factors are, amongst others, post-translational modifications as, for example, phosphorylation and acetylation [315,316], regulating assembly, organization, and function of intermediate filaments [317,318,319]. A further influencer of intermediate filament organization is the plakin family of proteins, connecting microtubules and actin to intermediate filaments [320]. Additionally, plakins connect intermediate filaments in desmosome adhesions and cell-matrix hemidesmosome adhesions to the actin and microtubule cytoskeleton and the nucleus [321,322] and intermediate filaments to one another [323]. Some intermediate filaments can also orient themselves along the actin or microtubule cytoskeleton. Actin and microtubules thereby form the guiding structures where filaments are transported along, either by kinesin and dynein (microtubules) or myosin (actin), resulting in a mutually dependent organization of intermediate filaments and actin and/or microtubules [232,324]. Interestingly, vimentin transport along microtubules can be inhibited by the actin meshwork, demonstrating a complex interaction between the cytoskeletal components [325]. Intermediate filaments are not only associated with other cytoskeletal proteins and (hemi-)desmosomes, but in the case of vimentin, it also binds via plectin or integrin α2β1 to actin and/or focal adhesions and promotes their strengthening [299,326,327,328]. A direct interaction between the vimentin tail domain and actin is also proposed [329]. The interactions of intermediate filaments discussed here and their interactions with microtubules and actin are summarized in Figure 2 and Figure 3 and Table 3.

Intermediate filaments also link the nucleus to the cytoplasmic cytoskeleton via the LINC (linker of nucleoskeleton and cytoskeleton) complex that is present at the nuclear membrane [330], binding to plectin [331] and, therewith, to intermediate filaments. Consequently, a disruption of LINC function leads to a disturbed force transmission [332,333] due to the weakened linkage of the nucleus and cytoskeleton. Similarly, depletion of nestin, vimentin, and GFAP in astrocytes leads to positional and rotational changes of the nucleus [304]. A correlation was found between nuclear rigidity, chromatin organization, and vimentin levels, indicating a crucial role of cytoplasmic intermediate filaments as passive mechanotransducers to the nucleus to control gene expression [334]. Similarly, GFAP mutations and changes in desmin organization alter gene expression [335].

Further types of intermediate filaments are the nuclear lamins. While nuclear lamins near the nuclear periphery form a filament network the organization of lamins in the center of the nucleus is only partly understood [336]. It was proposed that the nucleoplasmic lamins may form filaments, short fibrous structures, foci, or an unstructured “veil” [336]. The presence of seemingly less dense structures of lamins in the nucleoplasm is also in agreement with the observation of their higher mobility [337]. Interestingly, a lamin A knockdown inhibits the expression of actomyosin cytoskeletal related genes, as shown in mesenchymal stem cells [338]. Similarly, increased or reduced expression of lamin A inhibits or favors migration through a porous 3d matrix [339,340]. On a functional level, A-type lamins seem to impact mechanosensing and signaling [341,342] and to contribute to nuclear stiffness [343].

## 5. Involvement of Cytoskeleton in Cell Motility and Focal Adhesions

One highly important property of a cell is its ability to move, especially conceivable in the context of immune cells chasing pathogens, wound closure, or metastasis of tumor cells. For cells to move efficiently, a few universal steps are necessary, as follows: It needs to form protrusions that attach to its surroundings and, subsequently, a contraction and retraction of the rear is necessary [344]. A characteristic of cell migration is the precise coordination of these events in space and time. If, for example, the maturation of the adhesions at the cell front is not completed, an increase in contraction leads to the rupture of the newly formed adhesions, abandoning productive movement.

To achieve productive movement and the right timing of migration steps, cells have developed two distinct modes of migration, the amoeboid and mesenchymal type. Amoeboid cell migration is characteristic for rounded cells with low adhesion and high Rho-driven contractility, whereas mesenchymal migrating cells show strong adhesion and Rac1-induced protrusions [345], in line with the mutual negative regulation of Rac1 and RhoA [346,347].

### 5.1. Actin in Motile Processes

Actin filaments are the main contributors to cell migration in terms of force generation at the cell front and contraction at the rear. As stated, a cell can, in principle, use two types of migration, amoeboid and mesenchymal. While mesenchymal motion is mostly achieved via the extension of the lamellipodium or filopodia, the amoeboid migration works via the extension of blebs.

The lamellipodium is one of the main force generating cell structures, generating pushing forces of up to 35 nN in the extreme case of fish keratocytes [348,349]. To generate these forces, actin is polymerized locally at the cell front via Arp2/3 and depolymerized at the back of the lamellipodium by ADF/cofilin [350]. Interestingly, formins such as FMNL2 or FMNL3, seem to participate in lamellipodial extension independent of Arp2/3 complex incorporation and are, in some cell types, major sources of lamellipodial protrusion forces [351]. The protein Arpin inhibits the activity of Arp2/3, leading to pause phases in lamellipodial extensions and less directed motion [352]. The continuous (de-)polymerization of actin creates a treadmilling effect and consequently forces and retrograde flow of actin [76,77]. A flow in the opposite direction of the retrograde flow is generated by stress fiber contraction, transporting actin to the cell front [77]. To hold the lamellipodium in place and prevent retraction via e.g., actin cortex tension, the formation of new cell-ECM contacts is necessary. Generally speaking, during lamellipodium extension nascent adhesions, that mature into focal adhesions or disassemble, form [353]. While Rac1 controls the formation of nascent adhesions [354,355], maturation is controlled by RhoA and myosin II induced contractility [109], making them anchorage sites for stress fibers that generate tension and, thus, also control the composition of foal adhesions [356,357]. The exact actin nucleation mechanism in focal adhesions is only partly understood, but formins, such as FHOD1 or mDia1, are supposed to play a role [358,359]. The formation of nascent adhesions, in contrast, is thought to depend on Arp2/3 activity in the lamellipodium, due to its interaction with vinculin and focal adhesion kinases (FAK) [360,361]. Nevertheless, the lamellipodium is not essential for migration as several cell types, including fibroblasts and melanoblasts, can migrate without Rac or Arp2/3, but significantly slower [362,363,364]. In the absence of the lamellipodium, these cells migrate via filopodia or other, probably formin dependent, pseudo-pods [362,363,364]. For productive movement it is necessary to spatially restrict actin polymerization to one zone. Thus, it is assumed that Rac is only locally active. A mechanism to locally activate Rac1 is believed to function via Cdc42 induced pathways and microtubule capture at the leading edge and the subsequent local RacGEF and vesicle supply (see also next chapter) [365,366,367]. A further possible mechanism involves the Rho/ROCK pathway and actomyosin contractility to inhibit lamellipodium formation in multiple cell regions [368]. This idea is supported by the occurrence of multiple or larger lamellipodia after inhibition of Rho or ROCK activity [368], giving a strong hint to the importance of a finely tuned dynamic equilibrium between contractile and expansive forces. Although RhoA/ROCK is active at the cell front, a too high activity impairs lamellipodium based migration via retraction of the lamellipodium [369]. 

A second model, describing the generation of protrusions necessary for migration, is so-called blebbing. This mode of migration is observed in e.g., amoebae, tumor cells, neutrophils, or primordial germ cells or more generally in none- or weakly adherent cells, cells moving in a three dimensional matrix, or in confined environments [370]. As previously described, blebs are initially actin free structures that arise by hydrostatic pressure causing a detachment of the actin-cortex from the membrane, thus extending the cell membrane. Hydrostatic pressure is generated mostly by actin cortex contraction. Local RhoA activation inducing myosin activation leads to increased contractility. The so generated hydrostatic pressure causes bleb formation through hydrostatic flow [172,371,372,373]. Similarly to mesenchymal motion, cells using blebbing for migration need to “attach” the newly formed bleb to the surroundings and “detach” the cell rear, according to most models [370]. It seems that adhesion of cells using blebs for migration is very low [374,375], implying that strong cell adhesions may actually impede bleb-based motion. Consequently, both a low adhesion and high cortex contractility favor the amoeboid motion type [370]. One form of attachment of blebbing cells to their surroundings is via “chimneying” [376], which works via forces perpendicular to the direction of motion and, consequently, is independent of specific adhesion molecules. A different model proposed that forces are transmitted via cell-substrate intercalations. If blebs extend and form protrusions at the side of the cell into gaps of the substratum, then contractility of the reestablished cortex can than generate a net force to pull the cell body [377,378]. Despite these models, other forms of bleb based propulsion have been suggested, like a flow friction driven or a “swimming in low Reynolds numbers” model [370]. As bleb initiation and growth is mainly governed by myosin contractility and the actin cortex-membrane linkage, it is not surprising that the level of the actin-membrane cross-linker ezrin is increased at the cells rear and reduced at its front in carcinoma cells using blebbing for migration [181,379,380]. Similarly, increasing the level of other ERM proteins impedes bleb formation and bleb induced migration [172,381,382], while reducing ERM protein levels has the opposite effect [157,172,383]. A further factor that critically limits bleb extension and, thus, migratory properties is the cell membrane, that can usually stretch only about 4% before rupturing [384]. As blebs do not normally contain endosomes [169], it is suggested that bleb expansion is allowed by the local unfolding of the membrane. Furthermore, bleb expansion is faster than lamellipodial growth, can occur in arbitrary directions, and, because they do not contain the cortex, they can naturally adapt to three dimensional environments. Therefore, blebs might be of high importance in complex three dimensional (in vivo) environments, where lamellipodial extension is seriously impeded [345,385,386].

For efficient migration the cell rear needs to contract as well. To actively contract, actin structures use myosin to slide anti-parallel actin fibers along each other, creating contractile forces if the filaments are anchored at e.g., focal adhesions. The best studied contractile structures anchored to the substrate are stress fibers. Typically, stress fibers are directly linked to focal adhesions (except for transversal arcs), connecting the cell via actin fibers to the ECM [105,109]. Interestingly, the formed focal adhesions are stress dependent and inhibition of myosin II generated contractility decreases focal adhesion size [146], while external tension favors focal adhesion maturation [387]. Additionally, the forces acting on focal adhesions can lead to conformational changes of mechanosensitive proteins contained in focal adhesions, like β-integrins or talin [388,389,390], allowing stress fibers to convert mechanical into chemical signals, influencing focal adhesion maturation and turnover [391]. Therefore, dorsal stress fibers help the maturation of focal adhesions via tension at the leading edge and ventral stress fibers at the trailing edge [392]. Thus, stress fibers are highly important for cell adhesion but their function during cell migration remains poorly understood, as they are absent from many fast migrating cells, like leukocytes and *Dictyostelium discoideum* amoeba, as well as from cells embedded in soft three dimensional matrices [393,394]. Consequently, it was proposed that stress fibers are not necessary for migration. Under certain circumstances, they might have an inhibitory effect on migration because the turnover is comparably slow and contractile forces may impede cell motion [395]. Thus, the significance of stress fibers may be linked to their role in deforming the ECM, stabilizing focal adhesions, and through the generation of tension for rear contraction inside the cell [396]. For rear retraction it seems as if the contractile forces generated by ventral stress fibers are of importance for the disassembly of posterior adhesions and an inhibition of protrusions at the rear [397,398]. As stress fiber contractility in non-motile cells is associated with strengthening of focal adhesions, it needs to be tightly regulated to achieve just the right amount for the detachment of posterior adhesions. Consequently, a too strong RhoA activation inhibits cell migration via increased contractility [395,399] and inhibition of contractility via ROCK inhibition can even increase motility in some cell types under specific conditions [400,401]. The idea of rear retraction via stress fibers is further promoted by an adhesion gradient with lower adhesiveness at the rear [402].

Taken together, actin or, to be more precise, the lamellipodium, filopodia, and blebs are the main causes of force generation for cell motility and contractile structures like stress fibers or the actin cortex are drivers of rear contraction.

### 5.2. Microtubules in Motile Processes

In contrast to actin, microtubules are mostly not associated with force generation during migration, but rather with cell polarization and focal adhesions. The role of microtubules can, in principle, be divided into three categories, as follows: Participation in cell motility via their own mechanics, via signal transduction, and as a transport structure.

Microtubules are capable of bearing high external pressure and, thus, help to maintain the cells’ shape under physiological conditions [403]. In migrating cells, the microtubule (+)-end points in the direction of the plasma membrane and microtubules reaching the leading edge grow, at least in epithelial cells, more persistent [404], associated with EBs [216]. As discussed before EBs can recruit further +TIPs that promote microtubule stabilization, protrusion formation, and cell migration [212,405,406,407]. Other +TIPs, such as Clip-170 or its binding partner Clasp, act as rescue factors and increase the time of microtubules near the cortex [212,406]. Stabilization of growth can lead to a more persistent force transmission, even though the maximal pushing force decreases quadratically with length, due to buckling. Notably, the stabilization of microtubules does not only promote a more persistent microtubule growth, but also a steadier supply with material needed for migration, as these microtubules persist longer near the leading edge, being ideal tracks for material supply, in agreement with the preference of some kinesin motors for microtubules stabilized by acetylation and detyrosination [408]. Consequently, the polymerization of microtubules can generate a force of a few piconewton, on the same order as the force generated by motor proteins [161,263]. An in vitro study demonstrated that the generated forces can indeed deform membranes [409]. For a direct involvement of microtubules in the force generation process, a significant amount of microtubules actually have to reach the cell front. In most cell types, only very few microtubules reach the lamellipodium and the generated force is insufficient to generate large scale protrusions [410]. In contrast, in neurons and astrocytes, microtubules are capable of generating protrusions. In neurons, microtubules are also found unattached to centrosomes [411,412], forming bundles and generating large enough forces to participate in axon formation [410]. The free bundles (−)-ends are stabilized by members of the CAMSAP/Patronin family [413,414,415]. Notably, in axons these bundles point with their (+)-end away from the cell body [416], thus being capable of creating larger forces than single microtubules [268], sufficient to promote neurite outgrowth [417].

Cell motion associated structures have a high material consumption and, therefore, a steady supply is necessary to allow a continuous movement. For this transport, microtubules and their associated motor proteins are of high importance [226,227] because they can transport membrane components needed for membrane extension, signaling molecules, such as the small GTPases Rac and Cdc42, GEFs, and proteases, but also intermediate filaments and their precursors [230,231,418,419]. Additionally, microtubules transport β-actin coding mRNA and Arp2/3 subunits to the cell front [235,236].

A further indirect way for microtubules to influence cell motility is via the (de-)stabilization of cell-matrix adhesions or regulation of actin (de-)polymerization, as microtubules are associated with focal adhesions [420], their regulation [421], RhoGTPase activity [422], and, consequently, actomyosin contractility [423]. This can easily be seen by experiments with microtubule destabilizing agents like nocodazole, causing cell protrusion defects due to reduced Rac1 induced actin polymerization and increased contractility because of Rho-myosin II signaling [424,425]. This is supported by observations showing that microtubule growth can locally activate Rac1, favoring the generation of new focal adhesion sites [422] and the lamellipodium [425]. In neuronal cells, Rac might be activated by TIAM1 (T-cell lymphoma invasion and metastasis-inducing protein 1) interacting with microtubules via MAP1B [426]. An additional involvement of the Rac activators TRIO (triple functional domain protein) and TIAM2 was suggested in microtubule induced protrusion formation [422,427]. A RhoGEF possibly involved in microtubule dependent regulation of RhoGTPases is H1, which interacts with microtubules, is inactive when bound, and transitions into an active state when microtubules depolymerize [424,428,429,430,431].

Furthermore, microtubules grow in the direction of existing focal adhesions at the cell front where they get entrapped and stabilized [432] and accelerate the maturation of focal adhesion via the transport of integrins [433]. Thereby, stress fibers seem to function as a guidance structure for microtubules, mediated by MACF1 [279,434]. A positive feedback loop is also possible where integrin stimulation could cause a favored delivery of cargo at the site of adhesion [435]. A further possibility is an interaction of FAK or paxilin with APC that clusters at microtubule tips [436,437,438]. An additional signaling mechanism for APC and +TIPs, like Clip170 and CLASPs, is via the Rac and Cdc42 effector IQGAP and the formin mDia, promoting actin nucleation at focal adhesions [277,439,440]. Despite the adhesion favoring effect of microtubules, an opposing destabilizing effect was observed at the cell rear [432,441]. Microtubules actively targeted mature focal adhesions at the rear of motile fibroblasts, accelerating focal adhesion turnover [432,441]. A common model describes the phenomenon via the dynamic instability of microtubule filaments. By growing and targeting focal adhesions, microtubules exert a force on the adhesion site, depolymerize quickly afterwards, and repeat the process [442,443]. This hypothesis is supported by an observation showing a strong correlation between the microtubule poking number and the dissociation of focal adhesions [443]. However, how microtubules find and target focal adhesions is not yet fully understood. It has been observed that they grow along actin bundles towards adhesion sites, potentially cross-linking to actin via e.g., spectraplakins or others [213,444]. This idea is supported by the APC dependent localization of the spectraplakin MACF1 at the cell cortex, close to focal adhesions [445,446]. Additionally, in the absence of MACF1 peripheral microtubules are less well organized and adhesion turnover is inhibited [279]. Still, other mechanisms, involving CLASPs or interactions with integrin-linked kinase (ILK), are also possible [214,421,447,448]. Furthermore, the actin bundling protein fascin interacts with microtubules, promoting focal adhesion turnover via FAK [449]. Other mechanisms of microtubule dependent focal adhesion turnover are via clathrin mediated endocytosis of integrins, NBR1-mediated autophagy, or via vesicles carrying matrix metalloproteinases severing integrin-ECM connections [450,451,452,453].

### 5.3. Intermediate Filament Involvement in Motile Processes

It is well established that intermediate filaments crucially influence both cell-matrix adhesion and migration. Nevertheless, the precise mechanism of action of intermediate filaments is not fully elucidated. As for microtubules it seems as though intermediate filaments are not a direct part of the force generation mechanism necessary for movement, but rather a signaling platform and mechanical anchor inside the cytoplasm to transduce forces through the whole cell.

For this review we will focus the discussion mainly on vimentin, as vimentin is one of the best investigated intermediate filaments. Notably, we will not discuss keratins, as they are mostly restricted to epithelial cells and keratinocytes and not present in cell of glial origin. 

In non-migrating cells intermediate filaments are mostly localized around the nucleus, extending into the periphery [304,454], while they elongate into the lamella, connecting to focal adhesions near the leading edge in migrating cells [455,456]. In the lamellipodium intermediate filaments are found mostly in a non-filamentous state [326,454]. Generally speaking, intermediate filament organization alters the current state of the two other cytoskeletal components [457,458], thus potentially modulating both cell adhesion and migration.

A strong hint for intermediate filaments influencing cell migration comes from the observation that, for example, vimentin can interact with actin and neurofilaments with microtubules [329,459]. Vimentin is indeed necessary for motility of fibroblasts and breast cancer cells [460], epithelial cell wound closure [461], and other migration related phenomena [462]. Vimentin inhibition reduces motility in fibroblasts, astrocytes, and diverse cancer cells [463,464]. Studies evaluating possible mechanisms found evidence for vimentin directly binding to APC. APC regulates vimentin organization in astrocytes to align vimentin along the microtubule network [456]. Post-translational modifications, like detyronsination and acetylation of microtubules, also impact vimentin network organization [294,465]. On the other hand vimentin also affects polarized microtubule organization, amongst others by forming a template for microtubules, guiding microtubule growth, and, thus, favor directed migration [458,466]. The exact mechanism of this interaction is not yet fully understood but may be governed by APC, linking microtubules and vimentin or via vimentin phosphorylation [456,467]. Conversely, the vimentin filament network is dependent on microtubules and its motor proteins. Microtubule disruption leads to vimentin relocalization around the nucleus [234,468]. Furthermore, activation of Cdc42 during scratch-wound assay in astrocytes inhibits dynein mediated rearward transport of GFAP and vimentin containing filaments, promoting intermediate filament network extension in direction of the leading edge [469].

Another motility associated structure partly regulated by vimentin are focal adhesions. Vimentin is associated with formation, maturation, size, and strength of focal adhesions [300,313,326,327]. Vimentin regulates the Rac1 GEF VAV2 and its localization to focal adhesions to promote their stabilization via Rac1 induced FAK activation [470]. Depletion of vimentin in fibroblasts causes a FAK dependent induction of RhoA and myosin activity to compensate for the loss of tension induced by vimentin depletion [328]. Similarly, vimentin depletion leads to increased stress fiber assembly and myosin activity, via RhoA activation by activating RhoA GEF-H1, but without activating FAK in osteosarcoma cells [471]. Additionally, a triple silencing of vimentin, GFAP, and nestin or of vimentin and GFAP in astrocytes demonstrated that these intermediate filaments help to maintain the polarization of leader cells in collective motion by controlling forces in monolayers [464,472]. Silencing of each individual intermediate filament produced similar but less pronounced results [472]. This effect was attributed to larger and more focal adhesions that were distributed less concentratedly at the cell front [472]. It is supposed that those three intermediate filaments control focal adhesions and traction force in astrocytes via plectin to control vinculin recruitment [472]. In the case of vimentin, an interaction with integrin β3 was also observed [473]. A further hypothesis of how intermediate filaments control focal adhesions and traction forces is via the acto-myosin network, by redirecting forces and restraining actin retrograde flow [474], or by regulation of focal adhesions via microtubules [325,450,466,475]. Another mechanism that might explain the interaction of vimentin with focal adhesions is via the RAF-1/RhoA signaling [476,477], activating ROCK [478] and being able to phosphorylate vimentin, leading to a filament collapse and subsequent release of ROCK at the cells periphery [479]. Thus, the presence of vimentin can influence the RhoA signaling and, consequently, the formation and stability of focal adhesions [480,481].

In line with these findings, transverse arcs interact via plectin with vimentin to promote their retrograde flow and this coupling is necessary for the perinuclear organization of vimentin filaments [482]. Consequently, myosin II driven contractility of transversal arcs was made responsible for retrograde vimentin movement [482]. This is of special interest, as the local activation of Rac1 causes a local disassembly of vimentin at the lamellipodium forming side [454], also preventing polymerization of vimentin via the Rac1 and Cdc42 effector PAK [483]. Conversely, local vimentin depolymerization causes the formation of the lamellipodium at the side of vimentin depletion [454]. Consequently, vimentin co-regulates both contractile actomyosin bundles and protrusive lamellipodial actin, and thus cell polarization. Despite the regulation of actin dynamics in the lamellipodium and lamellum, vimentin seems to form a transport structure for the nucleus in a three dimensional environment (“nuclear piston”) in cooperation with actomyosin, via the creation of a pressure gradient [484].

Taken together, vimentin seems to mainly contribute to focal adhesion maturation and stabilization via the modulation of RhoA by FAK or Rho GEFs by maintenance of directed migration in collective motion and by regulation of the microtubule organization. Additionally, it seems as if vimentin locally suppresses the formation of the lamellipodium, favoring asymmetrical protrusion formation and, thus, generation of a net force.

There have also been studies of other intermediate filaments, but the understanding of their impact on cell migration and adhesion is limited. Here we will only give some non-exhaustive examples. Synemin is associated with migration in astrocytoma via interactions with the focal adhesion associated protein zyxin and regulation of actin dynamics [485,486]. Additionally, synemin was shown to interact with the focal adhesion components talin, vinculin, plectin, and α-actinin [283,487,488,489,490]. Nestin was demonstrated to regulate FAK, integrin α5β1 localization and VASP activity, controlling invasiveness of prostate cancer cells [491]. 

## 6. Cytoskeletal Alterations in Glioma

Malignancies belong to the most common causes of death worldwide, with increasing tendency [492,493]. Among them, brain tumors occur at a rate of 5–10 per 100,000 individuals [494]. The most frequent type of brain malignancies are gliomas that can be differentiated in astrocytoma, oligodendroglioma, and oligoastrocytoma. Especially high grade glioma are characterized by a highly infiltrative growth behavior, leading to low patient survival times [495,496,497]. The infiltration of adjacent regions of the brain is one of the most critical parts in patients’ survival, as it makes complete tumor resection almost impossible, consequently leading to frequent recurrences [495,496,498].

As migration and, thus, infiltration of glioma cells is largely governed by reshaping the cytoskeleton, it is no surprise that the composition and organization of the cytoskeleton in glioma cells differs strongly from that of healthy brain cells. In general, the Rho GTPases Rac1, Cdc42, and RhoA play a pivotal role in cell migration and cytoskeletal organization. The same is true for glioma cells, showing an increased expression of the mutually inhibiting GTPases RhoA and Rac1 [499]. Additionally, invading glioma cells exhibit an increased Cdc42 and Rac1 activity, but a decreased activity of RhoA at the cell front, held responsible for the enhanced migration of leader cells [500,501,502,503]. Similarly, cells at the invading front show a markedly increased FAK expression [504]. Thereby, the activity of RhoA seems to cause ambivalent effects. On the one hand, it is strongly expressed at the cell front and necessary for migration and, on the other hand, a marked activation causes an inhibition of glioma migration, via an inhibition of Rac1, and increased contractility, via larger focal adhesions [505,506]. Inhibition of the RhoA target ROCK led to increased migration in glioma cells via an activation of Rac1 [507] and Rac1 inhibition was associated with reduced migration and invasion [507,508,509]. Contrasting these observations, other studies found ROCK inhibition associates with the reduced migration of glioma cells [510,511]. A possible explanation for this discrepancy might be due to biphasic effects of ROCK activity. While low ROCK activity inhibits motion via low contractility and adhesion, a too high activity may impede migration due to contraction of protrusions and large adhesion sites, in a similar manner as proposed for the relation between adhesion and migration speed [512]. The mutual inhibition of RhoA and Rac1 is further complicated by Cdc42 being capable of activating Rac1 in glioma [513]. Interestingly, the formin mDia1, which is activated by RhoA, plays a major role in glioma cell polarization. For mDia1 expression, an association was demonstrated with the microtubule dependent localization of APC and Cdc42 at the cell front, regulating cell polarization [514]. The impact of RhoA and Rac1 on glioma migration is in good agreement with the observation of an increased FAK activation, negatively regulating RhoA and activating Rac1 in glioma, being associated with staging and a poorer prognosis [504,515]. In contrast, downregulation of FAK is associated with reduced migration [516,517]. The important role of RhoA points to myosin as a further important player in glioma migration. Indeed, myosin was found to be crucial for glioma cell migration in vivo, likely for rear retraction and nucleus deformation [518,519,520]. The lamellipodium is another structure associated with migration of glioma cells. Even though its main organizing protein Arp2/3 is associated with glioma staging, its exact role in glioma migration is not yet clear [521]. While Arp2/3 inhibition was reported to reduce lamellipodial size and migration in a scratch wound assay, another study recognized no such relationship on linear functionalized tracks [521,522]. Interestingly, Arp2/3 might also be involved in the maintenance of the glioma stem cell character [523]. When cortactin, an important cross-linker in the dendritic actin network with overexpression in glioma, is inhibited the size of the lamellipodium and scratch wound closure is reduced [524].

Silencing of the actin bundling protein fascin was demonstrated to reduce migratory capabilities of glioma cells, their division rate, and to increase the sensitivity to cytotoxic lymphocytes [525,526]. The drop in migration was attributed to a loss of filopodia after fascin-1 silencing [525,526].

Glioma cells are also capable of generating blebs for cell protrusion [527,528,529,530]. The switch from mesenchymal to amoeboid motion occurs under conditions of low adhesion, via e.g., integrin β1 blockade, p130Cas silencing, or high contractility via RhoA activation [527,528,529,530]. This is of special interest as migration capabilities of these cells was largely unchanged in 3D, pointing to an escape mechanism for interventions targeting “classical” migration associated molecules, such as e.g., integrin β1 or, potentially, others associated with mesenchymal migration [527,528,529,530]. Still, the exact role of amoeboid motion in glioma invasion remains largely unknown.

Glioma cells may express the intermediate filaments vimentin, GFAP, nestin, synemin, and α-internexin [531,532]. Studies evaluating the organization and expression of intermediate filaments in glioblastoma found the following three distinct subpopulations with: (1) High vimentin, GFAP, and synemin expression or (2) low vimentin, GFAP, and synemin expression or (3) high nestin but low vimentin, GFAP, and synemin expression [533]. Whether this expression pattern is associated with the origin of these tumors or has a distinct impact on migratory behavior is not yet elucidated. These patterns might alternatively be related with the molecular sub-classes of glioma (proneural, proliferative, mesenchymal). As opposed to healthy glia cells, glioma generally possess increased amounts of the intermediate filaments vimentin, nestin, and GFAP [534,535,536]. While the amount of vimentin and the glioma stem cell marker nestin showed a negative correlation with patient prognosis and/or glioma staging [537,538,539,540], an association of GFAP expression with staging is controversial. A recent meta-study reported about a missing correlation between the amount of GFAP and the grade of gliomas [536]. Interestingly, GFAP serum levels were proposed as a marker to differentiate between primary brain tumors and brain metastasis [541]. The α-internexin levels correlated negatively with staging, but only in oligodendroglioma [532]. These findings correlate well with the infiltrative or migratory phenotype obtained by glioma cells, as vimentin and nestin are associated with more motile characteristics (see before), while a forced expression of GFAP was shown to inhibit glioma motility in vitro [542,543]. Vimentin may additionally have a role in radiation induced migration of glioma cells. After non-lethal irradiation, the expression of vimentin was increased and associated with faster migration [544]. Furthermore, a nestin down-regulation is associated with an increased adhesion to collagen, fibronectin, and laminin, leading to an inhibition of migration of glioma cells, while overexpression had the reverse effect [545]. This might be of further interest, as nestin was especially found in cells at the invasive front of glioma [546]. The underlying molecular mechanisms of these nestin induced effects remained unclear so far. It remains tempting to speculate that, in glioma, a similar mechanism as discussed before might be responsible for the increased migratory capacity, via an activation of FAK and regulation of integrin localization [491]. The intermediate filament synemin seems to be an additional part of the motile machinery of astrocytoma cells. Synemin is co-localized with the actin cross-linker α-actinin at the cell front and down-regulation of synemin impaired astrocytoma motility, via reduced F-actin and α-actinin amounts [486,547]. Nevertheless, precise mechanisms of synemin actions in glioma are missing.

Intermediate filaments are not only differentially expressed in glioma, but β-III, β-IV, and γ-tubulin are also overexpressed in glioma [548,549,550]. Special attention should be given to β-III tubulin, which is not expressed in glia cells under physiologic conditions, but is up-regulated in high-grade glioma [551]. β-III tubulin might be responsible for the resistance of glioblastoma to the microtubule stabilizing agent taxol, as observed in carcinoma cells [552,553], but this concept is still under debate [551]. Notably, taxol was suggested to trigger differentiation in some glioma cells and in one study associated with an increase in GFAP expression [554,555]. The higher expression of γ-tubulin might be related to centrosome abnormalities, altered microtubule dynamics, and consequently, adhesion dynamics and cell polarization [556]. Furthermore, the microtubule severing protein spastin and the destabilizing protein stathmin are both associated with increased motility and, in case of spastin, also with reduced proliferation [557,558]. Spastin expression is increased in glioma and mainly located at the cell front and the mitotic spindle, implying an indirect role in both motility and cell division by destabilization of microtubules, inducing spindle formation defects during division and facilitating microtubule turnover at the front to adapt to changes of the microenvironment. For the microtubule associated protein DCX different, contradictory results were found. Few studies reported that DCX is preferentially expressed at the invasive front of glioma [559,560], indicating a pro-invasive role. Other authors demonstrated a very low DCX expression in glioma and that a forced expression causes apoptosis and inhibits invasion [561,562]. Accordingly, the role of DCX in glioma needs to be critically evaluated. In contrast, the role of EB1 seems to be unambiguous, the reduced accumulation of EB1 at microtubule tips is associated with higher microtubule instability, a less migratory and motile phenotype of glioma cells, caused by a lower amount of microtubules reaching the leading edge [563]. The microtubule stabilizing factor APC, despite regulating differentiation and cell cycle arrest [564,565], might also be part of the migratory machinery of glioma cells, as it was recently shown that vimentin, GFAP, and nestin organization, along microtubules in the glioblastoma cell line U138-MG, is critically dependent on APC [456]. APC may, additionally, increase the vimentin polymerization rate and, thus, influence migration [456]. The motor protein dynein is not differentially expressed in glioma cells in general, but migrating glioma cells express increased amounts of the protein [566]. Dynein is, in part, responsible for microtubule dependent intracellular transport and it can be speculated that dynein is necessary to maintain cell polarization by transporting inhibitory signals away from the cell front [567]. Similarly, kinesin-5 and microtubule-actin linker protein MACF1 are involved in increased migration and up-regulated similar to KiF2C and KiF14 in glioma cells [568,569,570,571]. The exact mechanism of MACF1 action is not elucidated yet, but a contribution of Wnt-signaling was proposed [571]. Further microtubule and actin-associated proteins are differentially expressed in glioma. A summary can be found in Table 4.

For productive migration, glioma cells generate an ECM differing from the normal micro-milieu of the central nervous system. This includes an overexpression of tenascin-C at the invasive front [590], increasing motility of glioma cells via interactions with β1 or αvβ3 integrins [591], probably via an activation of FAK and inhibition of RhoA [592,593,594]. The idea of such a mechanism is supported by the observed drop in glioma migration after inhibition of β1- or αv-integrin and increased motility after β1 overexpression [595,596,597,598]. In line with the association of integrins with migration is the elevated α-actinin expression in glioma, which is associated with a worse prognosis and connects focal adhesions to the cytoskeleton [577]. Despite tenascin-C other ECM components were also associated with a more migratory phenotype of glioma, such as laminin, fibronectin, and collagen [522,599,600], but myelin biomembranes are also efficient substrates for migration [601]. For thin linear laminin coatings trying to imitate properties of the basal lamina, a favored invasion route of glioma, it could be demonstrated that polarization of collective migration of glioma cells was microtubule dependent [522]. This migration mode could largely be inhibited by formin inhibition, but not by inhibition of Arp2/3 that led to an increased migration speed. This is in line with the general importance of formins for glioma migration, demonstrating that mDia antagonism/depletion and agonism/overexpression impaired migration and invasion [514,572]. The observed effect was, thereby, likely mediated via increased F-actin assembly and the stabilization of microtubules, leading to defects in protrusion formation, cell-cycle arrest, and apoptosis [572,602]. Hyaluronic acid/hyaluronan (HA), the main ECM component of the healthy brain, as well as their main receptors CD44 and RHAMM (receptor of hyaluronan-mediated motility) are also upregulated in glioma [603,604,605,606], but their exact role is not yet determined. In general, increased HA expression is associated with an increase in motility and migration of glioma [607,608], probably via a CD44 or RHAMM induced activation of Rho GTPases, such as Rac1 or Cdc42 [609,610,611]. Even so, the role of CD44 remains ambivalent in glioma cell migration. On the one hand low and high levels of CD44 are associated with a less migratory phenotype and on the other hand intermediate expression levels facilitate migration [612]. This biphasic effect is probably related to the substrate adhesiveness.

## 7. Conclusions

The actin and microtubule cytoskeleton, their interactions, and relation to cell migration are comparably well understood for healthy cells, but the impact of their interplay with intermediate filaments just started to gain attention. Consequently, the role of intermediate filaments during migration is less well understood and many questions, especially regarding the role of GFAP, vimentin, synemin, and nestin in glia cells, remain open. For malignant cells the situations worsen, due to altered expression and localization profiles of cytoskeleton associated proteins, leading to changes in the global organization of the cytoskeleton, the signaling, and turnover of structures. Additional attention should be paid to the heterogeneity of glioma, showing significant differences in expression profile and behavior between “tumor-core” and “invading” tumor cells, but also between glioma of different patients. In case of glioma, the impact of cytoskeletal variations is only sparsely investigated and will need a significantly increased amount of data to understand the migration of glioma in the central nervous system. This includes the effect of the observed overexpressions, like spastin, vimentin, nestin, actin cross-linkers, etc., on migration and the underlying mechanisms, but also how migration associated signaling is altered in glioma and whether this can be used for specifically targeting glioma migration. Furthermore, the function of stress fibers in glioma migration and whether blebs play a significant role during brain infiltration by glioma are also not yet addressed adequately. Current approaches targeting the cytoskeleton in glioma mainly focus on microtubules, but mostly not to alter migratory properties but to induce cell cycle arrest or apoptosis. Nevertheless, these approaches remained futile because of glioma heterogeneity, therapeutic side effects, or resistance mechanisms. 

## Figures and Tables

**Table 1:** Summary of mentioned actin associated proteins and their direct or indirect functions.

Actin Associated Proteins	Function
Arp2/3	Polymerization factor
Ena/VASP	Polymerization factor, anti-capping function
FMNL2	Polymerization factor
mDia1	Polymerization factor
mDia2	Polymerization factor
Profilin	Inhibits actin polymerization
ADF/Cofilin	Actin severing
Arpin	Inhibits Arp2/3
Myosin II	Cell/actin contractility, cross linker
RLC	Activates myosin II
MLCK	Activates myosin II
MHCK	Inhibits myosin II activity
PKC	Inhibits myosin II activity
CKII	Inhibits myosin II activity
Scruin	Cross linker
Fascin	Cross linker
α-actinin	Cross linker
Filamin	Cross linker
Fimbrin	Cross linker
Paladin	Cross linker
Ezrin	Membrane-cortex linker
Radixin	Membrane-cortex linker
Moesin	Membrane-cortex linker
Cdc42	Signaling molecule, activates mDia2, WAVE, N-WASP
Rac1	Signaling molecule, activates WASP/WAVE, arpin
RhoA	Signaling molecule, activates ROCK, mDia1, LIMK
ROCK	Signaling molecule, activates myosin II
WASP/WAVE	Signaling molecule, activates Arp2/3
N-WASP	Signaling molecule, activates Arp2/3
LIMK	Signaling molecule, inhibits ADF/cofilin

**Table 2:** Summary of mentioned microtubule associated proteins and their direct or indirect functions.

Microtubule Associated Proteins	Function
Stathmin	Depolymerization
XMPA215	Polymerization factor
EB	Polymerization, Stabilization, Recruitment of proteins
DCX	Polymerization factor, Stabilization
CLASP	Stabilization
APC	Stabilization
mDia1	Stabilization
mDia2	Stabilization
Tau	Stabilization
MAP2	Stabilization
Spastin	Microtubule severing
Katanin	Microtubule severing
Kinesin	Cargo transport
Dynein	Cargo transport
MACF1	Actin-Microtubule interactions
Cdc42	Signaling molecule, activates PAK
Rac1	Signaling molecule, activates PAK
RhoA	Signaling molecule, mDia1
PAK	Signaling molecule, inhibits stathmin

**Table 3:** Summary of mentioned intermediate filament associated proteins and their function.

Intermediate Filament Associated Proteins	Function
LINC	Nucleus—intermediate filament linkage
Plakins	Linkage to adhesion sites
Plectin	Intermediate filament—integrin linkage
Kinesin *	Filament transport
Dynein *	Filament transport
Myosin *	Filament transport

* Involved in the transport of filaments as their cargo.

**Table 4:** Summary of motility associated proteins differentially expressed in glioma, compared to healthy glia cells.

	Function	Expression/Activity	Sources
Actin associated proteins			
Arp2/3	polymerization	high	[521]
mDia2 (formin family)	polymerization	high	[572]
Profilin	polymerization	low	[573]
Moesin/Ezrin	membrane to actin cortex linkage	high	[574,575,576]
Cortactin	actin cross-linker	high	[524]
Filamin	actin cross-linker	high	[548]
α-actinin	actin cross-linker	high	[577]
Fascin	actin cross-linker	high	[578,579]
**Microtubule associated proteins**			
MAP2	stabilization	high	[580,581]
Sclip (Stathmin family)	destabilization	high	[582]
Spastin	destabilization	high	[557]
MACF1	microtubule-actin linkage	high	[571]
Dynein	cargo transport	unchanged	[566]
Kinesin-5, KiF2C, KiF14	cargo transport	high	[568,569,570]
Β-III, β-IV, γ tubulin	microtubule formation and anchorage	high	[548,549,550]
**Intermediate filaments**			
Vimentin/Nestin	cytoskeletal meshwork	high	[538,539,545,546,583,584,585]
GFAP	cytoskeletal meshwork	high	[536]
α-Internexin	cytoskeletal meshwork	high	[532]
**Signaling molecules**			
RhoA	contractility	high/low	[499,586]
RhoB	contractility	low	[586]
RhoG	contractility, protrusion formation	high	[587]
Rac1	protrusion formation	high	[499]
FAK	protrusion formation, adhesion turnover	high	[504,588,589]
